# Impact of the HEAD-US Scoring System for Observing the Protective Effect of Prophylaxis in Hemophilia Patients: A Prospective, Multicenter, Observational Study

**DOI:** 10.4274/tjh.galenos.2021.2020.0717

**Published:** 2021-06-01

**Authors:** Kaan Kavaklı, Süha Süreyya Özbek, Ali Bülent Antmen, Fahri Şahin, Şevkiye Selin Aytaç, Alphan Küpesiz, Bülent Zülfikar, Mehmet Sönmez, Ümran Çalışkan, Can Balkan, Tuğana Akbaş, Taner Arpacı, İpek Tamsel, Turgut Seber, Berna Oğuz, Can Çevikol, Mesut Bulakçı, Polat Koşucu, Demet Aydoğdu, İlgen Şaşmaz, Gülen Tüysüz, Başak Koç, Hüseyin Tokgöz, Zuhal Mehrekula, Burcu Özkan

**Affiliations:** 1 Ege University Children’s Hospital, Clinic of Children’s Health and Diseases, Division of Pediatric Hematology, İzmir, Turkey; 2 Ege University Medical Faculty Hospital, Clinic of Radiology, Division of Hematology, İzmir, Turkey; 3 Acıbadem Adana Hospital, Clinic of Pediatric Hematology, Adana, Turkey; 4 Ege University Medical Faculty Hospital, Clinic of Internal Diseases, Division of Hematology, İzmir, Turkey; 5 Hacettepe University Faculty of Medicine, Department of Children’s Health and Diseases, Division of Pediatric Hematology, Ankara, Turkey; 6 Akdeniz University Hospital, Clinic of Children’s Health and Diseases, Antalya, Turkey; 7 İstanbul University Hemophilia Comprehensive Care Center, İstanbul, Turkey; 8 Karadeniz Technical University Medical Faculty Farabi Hospital, Clinic of Internal Diseases, Division of Hematology, Trabzon, Turkey; 9 Necmettin Erbakan University Meram Medical Faculty Hospital, Department of Children’s Health and Diseases, Konya, Turkey; 10 Acıbadem Adana Hospital, Clinic of Radiology, Adana, Turkey; 11 Hacettepe University Faculty of Medicine, Department of Radiology, Ankara, Turkey; 12 Akdeniz University Hospital, Clinic of Radiology, Antalya, Turkey; 13 Karadeniz Technical University Medical Faculty Farabi Hospital, Clinic of Radiology, Trabzon, Turkey; 14 Necmettin Erbakan University Meram Medical Faculty Hospital, Clinic of Radiology, Konya, Turkey; 15 Pfizer Pharmaceuticals, Rare Disease Department, İstanbul, Turkey

**Keywords:** Hemophilic arthropathy, Joint Scores, HJHS, Ultrasonography

## Abstract

**Objective:**

This study aimed to observe the preventive effect of prophylactic treatment on joint health in people with hemophilia (PwH) and to investigate the importance of integration of ultrasonographic examination into clinical and radiological evaluation of the joints.

**Materials and Methods:**

This national, multicenter, prospective, observational study included male patients aged ≥6 years with the diagnosis of moderate or severe hemophilia A or B from 8 centers across Turkey between January 2017 and March 2019. Patients were followed for 1 year with 5 visits (baseline and 3^rd^, 6^th^, 9^th^, and 12^th^ month visits). The Hemophilia Joint Health Score (HJHS) was used for physical examination of joints, the Pettersson scoring system was used for radiological assessment, point-of-care (POC) ultrasonography was used for bilateral examinations of joints, and the Hemophilia Early Arthropathy Detection with Ultrasound (HEAD-US) score was used for evaluation of ultrasonography results.

**Results:**

Seventy-three PwH, of whom 62 had hemophilia A and 11 had hemophilia B, were included and 24.7% had target joints at baseline. The HJHS and HEAD-US scores were significantly increased at the 12^th^ month in all patients. These scores were also higher in the hemophilia A subgroup than the hemophilia B subgroup. However, in the childhood group, the increment of scores was not significant. The HEAD-US total score was significantly correlated with both the HJHS total score and Pettersson total score at baseline and at the 12^th^ month.

**Conclusion:**

The HEAD-US and HJHS scoring systems are valuable tools during follow-up examinations of PwH and they complement each other. We suggest that POC ultrasonographic evaluation and the HEAD-US scoring system may be integrated into differential diagnosis of bleeding and long-term monitoring for joint health as a routine procedure.

## Abstract (tr)

**Amaç:**

Bu çalışmada hemofili hastalarında profilaksinin eklem sağlığı üzerindeki koruyucu etkisini gözlemlemek ve eklemlerin klinik ve ultrasonografik olarak değerlendirilmesinin öneminin araştırılması amaçlandı.Amaç: Bu çalışmada hemofili hastalarında profilaksinin eklem sağlığı üzerindeki koruyucu etkisini gözlemlemek ve eklemlerin klinik ve ultrasonografik olarak değerlendirilmesinin öneminin araştırılması amaçlandı.

**Gereç ve Yöntemler:**

Ulusal, çok merkezli, prospektif ve gözlemsel olan araştırma Ocak 2017 - Mart 2019 arasında 8 ayrı merkezde takip edilen erkek ve 6 yaşından büyük orta seviyede veya ağır hemofili-A ve hemofili-B hastalarını kapsıyordu. Hastalar 1 yıllık takip sırasında toplam 5 ayrı vizitte (başlangıç, 3. ay, 6. ay, 9. ay ve 12. ay) değerlendirildi. Hemofili Eklem Sağlığı Skoru (HJHS) eklemlerin fiziksel bakısında kullanılırken Petterson skorlama sistemi radyolojik bakı sırasında değerlendirildi. Ayrıca, hasta başı ultrasonografisiyle bilateral eklem incelemeleri yapılarak HEAD-US skorlama yöntemiyle eklem skorları belirlendi.

**Bulgular:**

Çalışmada 62’si hemofili A ve 11’I hemofili B olan 73 hastanın %24,7’sinde hedef eklem varlığı mevcuttu. HJHS ve HEAD-US skorlarının tüm hastalarda takibin 12. ayında anlamlı olarak arttığı gözlendi. Hemofili A hastalarında daha yüksek skorlar saptandı. Skor artımı alt gruplarda değerlendirildiğinde çocukluk yaş grubunda artışın anlamlı olmadığı gözlendi. Başlangıçta ve 12. ayda yapılan üçlü bakıda HJHS, HEAD-US ve Petterson skorlarının anlamlı olarak korele olduğu saptandı.

**Sonuç:**

HJHS eklem skoru ile HEAD-US radyolojik skorlamasının hemofili hastalarının eklem sağlığının takibinde çok değerli olup birbirlerini destekledikleri yakından gözlendi. Hasta başı US skorlama sistemlerinin günümüzde hemofilide eklem sağlığının rutin takip ve izlemi sürecinde hem kanama ayırıcı tanısı hem de uzun dönemli takip açısından oldukça değerli bir yeri olacağını düşünüyoruz.

## Introduction

The goal of hemophilia treatment is to prevent bleeding by replacement of factor concentrates or substitution of missing coagulation factors. Coagulation factors can be given for prophylaxis (primary and secondary prophylaxis, etc.) or for treatment when needed [1]. Prevention of bleeding via prophylaxis is considered the gold-standard treatment in cases of severe hemophilia [1,2]. Provision of early prophylaxis for severely hemophilic children can completely or largely prevent life-threatening bleeding, chronic joint diseases, and disabilities; thus, requirements for surgical interventions can be decreased and both health and social well-being of people with hemophilia (PwH) can be improved [1,2,3].

Evaluation of joint status is crucial not only for staging joint disease but also for the follow-up of prophylaxis and for evaluating outcomes of replacement therapy. Joint function is widely assessed by the Hemophilia Joint Health Score (HJHS), but implementation of the HJHS requires training and experience. The Pettersson scoring system, a radiological joint scoring system, gives quite reliable outcomes when applied by an experienced radiologist [4,5]. Magnetic resonance imaging (MRI) is a more sensitive imaging method than plain radiogram in evaluating the joints. Nevertheless, MRI has disadvantages such as long scanning period, high cost, limited availability, and need for sedation in young children [4,5]. However, ultrasonography may provide advantages such as appropriate cost, availability, repeatability, faster examination, no need for sedation while examining children, and scanning of multiple joints and dynamic examination of joints in a single session.

The present study aimed to observe the preventive effect of prophylactic treatment on joint health in PwH and to investigate the importance of integration of point of-care (POC) ultrasonographic examination into clinical and radiological evaluation of the joints.

## Materials and Methods

### Patients

The current study was designed as a national, multicenter, prospective, non-interventional, observational study. A total of 8 centers across Turkey were selected and the data were collected from January 2017 through March 2019. Male patients aged ≥6 years with the diagnosis of moderate or severe hemophilia A or hemophilia B (factor level <2%) were included in the study. The pediatric group (47.9%) ranged in age from 6 to 18 years while the adult group (52.1%) ranged in age from 19 to 70 years (Table 1).

Patients with communication difficulties (unable to understand or speak Turkish) or cognitive dysfunction and patients with inhibitors were excluded. The study was approved by the Clinical Research Ethics Committee of the Ege University Faculty of Medicine and written informed consent was obtained from the patients or their legal representatives.

### Procedure

After enrollment in the study, the patients were followed for 12 months with a total of 5 visits (baseline and 3^rd^, 6^th^, 9^th^, and 12^th^ months). In the study centers, patient data concerning demographic features and hemophilia history were recorded on case report forms at the baseline visit. Physical examinations of elbow, knee, and ankle joints were performed with the HJHS during each visit. Annual bleeding rate (ABR) was recorded every 3 months during clinical visits.

Additionally, ultrasonographic examinations of the bilateral elbow, ankle, and knee joints were performed during each visit. Ultrasonography results of the patients were evaluated with the Hemophilia Early Arthropathy Detection with Ultrasound (HEAD-US) scoring system. Evaluations were performed by expert physiotherapists and radiologists. The same experts in each center performed the evaluations at different time points to prevent variability.

For all patients, quality of life (QoL) questionnaires were administered at the baseline, 6^th^ month, and 12^th^ month visits.

Compliance of prophylaxis and bleeding episodes were recorded every 3 months in clinical visits.

### Measurements

The HJHS is a scoring system used to assess physical joint damage in PwH and recommended for routine follow-up assessments of joint health. Using the HJHS, the six most commonly involved joints (elbows, knees, and ankles) are evaluated in PwH and total scores are within the range of 0-124. High scores indicate damage/impairment [6].

The Pettersson scoring system allows detailed evaluation of radiological changes in the joints. Posterior-anterior and lateral X-ray images of the joints are evaluated. Scores range from 0 to 13 for a single joint and the maximum possible total score is 78 when 6 joints are evaluated [7,8].

The HEAD-US scoring system was developed by Martinoli et al. [9]. It is based on three markers for the three main sets of joints (knees, elbows, and ankles): synovitis (score of 0-2), cartilage (score of 0-4), and subchondral bone (score of 0-2), with a maximum score of 8 points per joints.

The Short Form-36 (SF-36) is a questionnaire widely used to assess health-related QoL. Based on the scores of 8 dimensions of health (physical functioning, bodily pain, role limitations due to physical health problems, role limitations due to personal or emotional problems, general mental health, social functioning, energy/fatigue or vitality, and general health perceptions), two component summaries (physical component summary and mental component summary) are obtained. Higher scores indicate better health status [10].

The EuroQol-5 Dimension (EQ-5D) is another questionnaire frequently used to assess health-related QoL. Five parameters of health (mobility, self-care, usual activities, pain/discomfort, and anxiety/depression) are evaluated. Higher scores indicate better QoL [11]. In the present study, the three-level version (EQ-5D-3L) was used.

Patients receiving prophylaxis were evaluated using scoring systems and QoL parameters. Prospective evaluations of patients were provided for the one-year observational period.

Bleeding frequency and target joint availability are main reasons for starting prophylaxis for patients with moderate hemophilia. They were mostly receiving secondary prophylaxis.

Children below 10 years of age were in the primary prophylaxis group, whereas older children (10-18 years) received secondary prophylaxis. Most of the adult group received secondary or tertiary prophylaxis. Primary prophylaxis was started at once weekly and then increased to twice and thrice weekly. Most secondary prophylaxis patients received infusions twice or thrice weekly.

### Sample Size

The sample size was calculated as 80 PwH with the assumptions of the rate of damaged joints being 8% in PwH receiving prophylactic treatment and the rate of damaged joints being 25% in PwH not receiving prophylactic treatment, with 80% power when the statistical significance level was presumed as 0.05.

### Statistical Analysis

Data analysis was performed using PASW Statistics for Windows, Version 18.0 (SPSS Inc., Chicago, IL, USA). For descriptive statistics, numerical variables were expressed as mean, standard deviation, median, and minimum-maximum and categorical variables were expressed as number and percentage. Student’s t-test was performed in comparisons of ultrasonography scores and QoL scores at each visit when the condition of normal distribution was fulfilled. The Mann-Whitney U test was used when the condition of normal distribution was not fulfilled. For normally distributed data, repeated measures analysis was performed for the comparison of change in ultrasonography scores and QoL scores with time in the groups. When the normal distribution condition was not fulfilled, the Friedman test was performed separately in the groups. Spearman’s rho correlation test was used for correlation analysis.

The statistical significance level was set at p<0.05.

## Results

The present study included 78 male hemophilic patients, of whom 73 received prophylactic treatment and 5 received on-demand treatment in case of bleeding. The 73 patients receiving prophylaxis were included in the analyses. Of those patients, 62 had hemophilia A (58 severe and 4 moderate) and 11 had hemophilia B (10 severe and 1 moderate). General characteristics of the patients and disease-related data are presented in Table 1.

Among the included patients, 24.7% had target joints at baseline. The most commonly affected joint was the right ankle (27.4%). Overall, 31.5% of the included patients underwent major or minor surgical procedures and radioactive synovectomy was performed for only 6 patients. More than half of the patients were not able to go to work because of hemophilia in the last 3 months (Table 1).

The ABRs for all patients, for children, and for adults are shown in Table 2. The elevation of ABR rates was not significant for children or for adults.

Moreover, joint scores of all patients at the baseline and follow-up visits are shown in Tables 3, 4, 5, 6. When the scores at the baseline and 12^th^ month were compared, there were significant increases in both the HJHS and HEAD-US scores at the 12^th^ month in all patients. Separating the patients by age, no significant increase in any of the scores was observed in children, but significant increases in the HJHS, HEAD-US, and Pettersson scores were seen in the adult group.

Evaluation of the patients in terms of hemophilia A and hemophilia B subgroups showed significant increases in the HJHS and HEAD-US scores at the 12^th^ month in the hemophilia A subgroup (Tables 3 and 6). However, the number of hemophilia B patients included in this study is much smaller in comparison to the hemophilia A subgroup. For subgroup analysis by prophylaxis groups, results are shown in Tables 4 and 5. The elevated scores found in the adult group were statistically significant compared to the children.

Correlation analyses were performed for the joint scores at the baseline and 12^th^ month. Among all patients, the HJHS total score was found to significantly correlate with the HEAD-US total score at the baseline and 12^th^ month. Moreover, there was a significant correlation between the Pettersson total score and the HEAD-US total score at baseline and the 12^th^ month (Table 7). Strong correlation was found among the three scoring systems (HJHS, HEAD-US, and Pettersson) at baseline and after 12 months of observation (p<0.001).

There were no significant differences in the QoL scores between the baseline and 12^th^ month in all patients.

Neither pediatric vs. adult group nor hemophilia A vs. hemophilia B comparisons were significant.

Comparisons of the QoL scores of the patients between the baseline and 12^th^ month are presented in Table 8.

## Discussion

In all patients, there were significant increases in the HEAD-US and HJHS scores during the one-year follow-up period despite prophylaxis. This was consistent with the results obtained from the study by Warren et al. [12], in which only a small number of PwH had no damage in their joints when they reached adolescence, in spite of early prophylaxis. In the present study, subgroup evaluation according to age revealed a significant increase in the joint scores from baseline to the 12^th^ month in the adult group but not in the pediatric group. In other words, prophylactic treatment slowed down the progression upon onset of arthropathy in children, but degenerative arthropathic changes persisted in adult patients. Subgroup analysis results supported the importance of early prophylaxis in childhood.

Similarly, Warren et al. [12] reported that the risks of osteochondral damage on MRI and increased ABR and joint ABR were significantly higher in the children who began using prophylactic factor VIII after 6 years of age compared to children for whom prophylaxis was initiated before 2.5 years of age. These results show the importance of preventing initiation of joint damage by means of prophylaxis at an early stage in life, because the earlier the damage occurs, the more difficult it is to prevent its progression. For this reason, examination of the joints by means of regular follow-up is important for early detection and prevention of arthropathic changes in PwH. Geraghty et al. [13] showed that nonadherence to treatment was higher among adults compared to children with hemophilia. As adolescent and adult patients are prone to failures to adhere to clinical practice, we used reminders about compliance every 3 months during clinical visits.

Early initiation of prophylactic treatment and good adherence to treatment are important factors to reduce the incidence of bleeding and to preserve joint functions in PwH [14,15].

Hemophilic arthropathy is a progressive condition and negatively impacts the QoL of patients as damage progresses. It has been reported that QoL is lower in PwH as compared to the general population [16]. Earlier studies have demonstrated poorer QoL in patients with severe joint problems [16,17,18]. These data support the importance of appropriate treatment to limit joint injury. In the present study, QoL was assessed using the EQ-5D-3L and SF-36 questionnaires. The QoL scores of the patients were generally high, indicating a better QoL, and the mean scores were similar at the baseline and 12^th^ month. Assessment of QoL not only gives information about clinical statuses of patients but also provides an objective criterion for measuring benefit gained from treatment [19,20]. Better adherence of patients to treatment is associated with better outcomes and is a factor that improves QoL [21,22,23].

During the follow-up of PwH, joint function is assessed by physical examination and the HJHS is widely used for scoring. However, implementation of the HJHS requires training and experience [24]. Plain radiographs have also long been used to evaluate the musculoskeletal system. The Pettersson scoring system, a radiological joint scoring system, gives quite reliable outcomes when applied by an experienced radiologist [24]. MRI is a more sensitive imaging method than plain radiogram in evaluating the joints. Nevertheless, MRI has practical disadvantages such as the long scanning period, high cost, limited availability, and need for sedation in young children [24]. There is a need for an easy, cost-effective, repeatable, efficient, and reliable joint scoring system. Therefore, ultrasonography has become an attractive method in the last years for objective evaluation of joint status and for early detection of changes during periodic follow-up [25,26,27,28,29]. Ultrasonography provides advantages such as appropriate cost, availability, repeatability, faster examination, no need for sedation while examining children, and scanning of multiple joints and dynamic examination of joints in a single session [30]. Ultrasonography allows detection and quantitation of signs of disease activity (fluid collection in the joint, synovial hypertrophy, etc.) and degenerative cases (osteochondral changes, etc.) and it is also beneficial in discriminating inflammatory (serous) effusion from hemarthrosis. Ultrasonography, as a simple and practical tool, is a powerful potential tool to be utilized in routine hemophilia care in the near future [28].

Several scoring systems have recently been proposed to provide objectivity in ultrasonographic evaluation; the HEAD-US is one of them [28]. The HEAD-US scoring system has advantages such that it can be applied by non-imaging specialists. However, even though this scoring system can be performed by non-radiologists after a short training period, the speed of the exam is dependent on the sonographer’s experience level [24,31].

There are studies using the HEAD-US scoring to assess joint status in PwH and evaluating its correlation with HJHS scores. In a study evaluating joint status in children with severe hemophilia A receiving prophylaxis, 85.3% of the joints were found normal by the HJHS, whereas 79.0% of the joints were found normal by the HEAD-US scoring system [32]. While there was a correlation between the HEAD-US scores and bleeding scores, no correlation was determined between the HEAD-US and HJHS scores. Nevertheless, the HJHS and HEAD-US scores were concordant for 73.4% of the joints. Ultrasound detected minimal changes in 19.6% of the joints with normal physical functioning, whereas 12.2% of the joints that were considered normal on ultrasound showed changes according to the HJHS. As a consequence, ultrasound detected a higher percentage of abnormalities than physical examination [32]. Jiménez-Yuste et al. [33] carried out a study of hemophilia B patients and concluded that using the HEAD-US scoring system in routine practice provided patients with better and more objective evaluation and contributed to personalization of treatment. Li et al. [34] determined a significant correlation between the HEAD-US and HJHS scores in PwH receiving prophylactic treatment. Banchev et al. [35] reported a strong correlation between three-year joint bleeding rates and HEAD-US total scores for ankle and knee joints in hemophilia A patients receiving secondary/tertiary prophylaxis. Plut et al. [36] conducted a study of patients with severe hemophilia and determined a very high correlation between the overall HEAD-US scores and overall International Prophylaxis Study Group MRI scores. They suggested the HEAD-US protocol as a fast, reliable, and accurate method for detecting hemophilic arthropathy and determining its degree. In the present study, considering all patients, the HEAD-US total scores showed a correlation with both the HJHS total score and the Pettersson total score. Joint tissue activity and damage examination (the JADE protocol) was developed for soft tissue and osteochondral measurements for a POC ultrasound scoring system in the United States, patented by the University of California-San Diego [37]. The JADE protocol has similar principles, is easy to learn and administer, and is ideal for use in routine practice as well as achieving useful outcomes as a research tool. Both protocols (JADE and HEAD-US) appear feasible for quantifying hemophilic intraarticular abnormalities with lower variabilities.

### Study Limitations

The main limitation of the present study was not including more patients receiving on-demand treatment and thereby not comparing the data of patients receiving prophylactic treatment with those receiving on-demand treatment. Another limitation was missing some patients during the prospective evaluation due to data deficiencies. Even though we were able to calculate statistical analysis, without any missing patients we would have been able to provide even better results.

## Conclusion

The HEAD-US and HJHS scoring systems are valuable tools during follow-up examinations of patients and they complement each other. We suggest that POC ultrasonographic evaluation and the HEAD-US scoring system may be integrated into differential diagnosis not only for bleeding and but also for long-term monitoring of joint health of PwH as a routine procedure. It would thereby be possible to provide PwH maximum benefit by means of early diagnosis of joint changes and bleedings that might be overlooked during physical examination and, in turn, to personalize prophylactic treatment.

## Figures and Tables

**Table 1:**
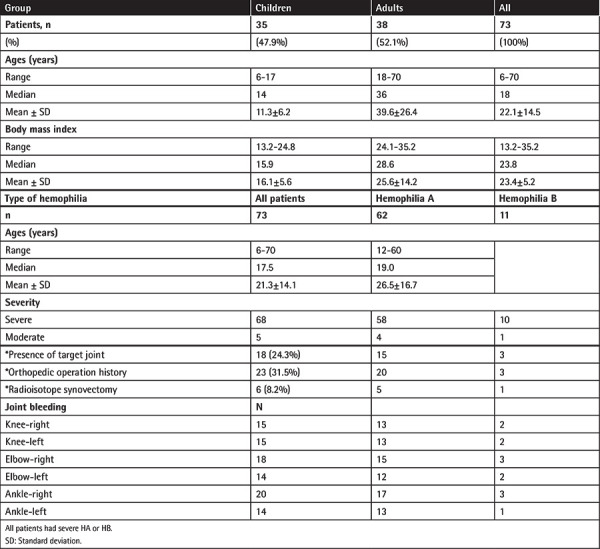
Demographics of 73 patients receiving prophylaxis.

**Table 2:**
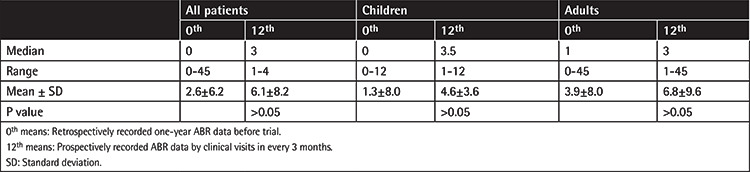
ABR rates for all patients and children and adults.

**Table 3:**
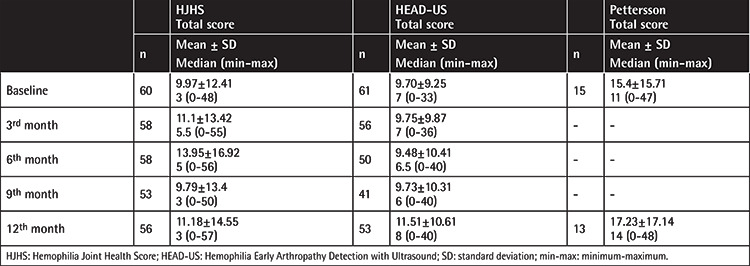
Joint scores of all patients at baseline and follow-up visits.

**Table 4:**
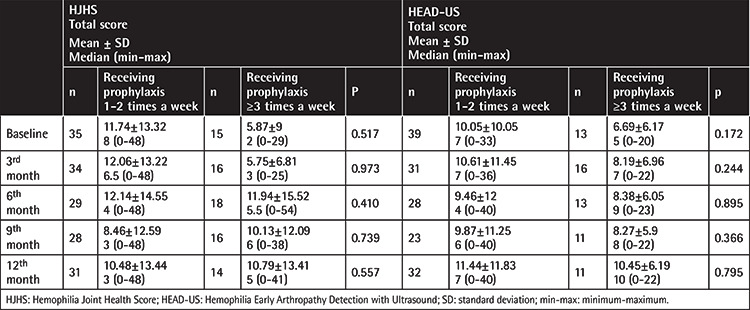
Both scoring systems and relationship with frequency of prophylaxis.

**Table 5:**
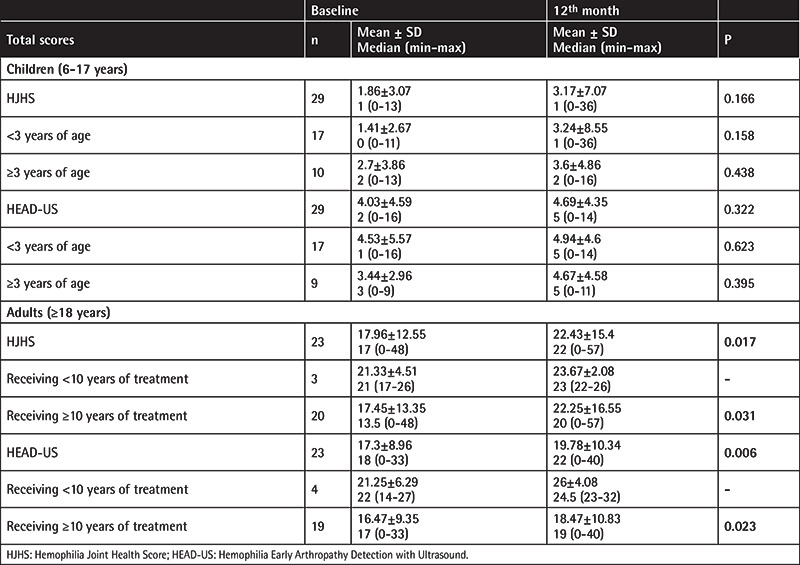
Prophylaxis regimens and relationship with scores in subgroups of children and adults.

**Table 6:**
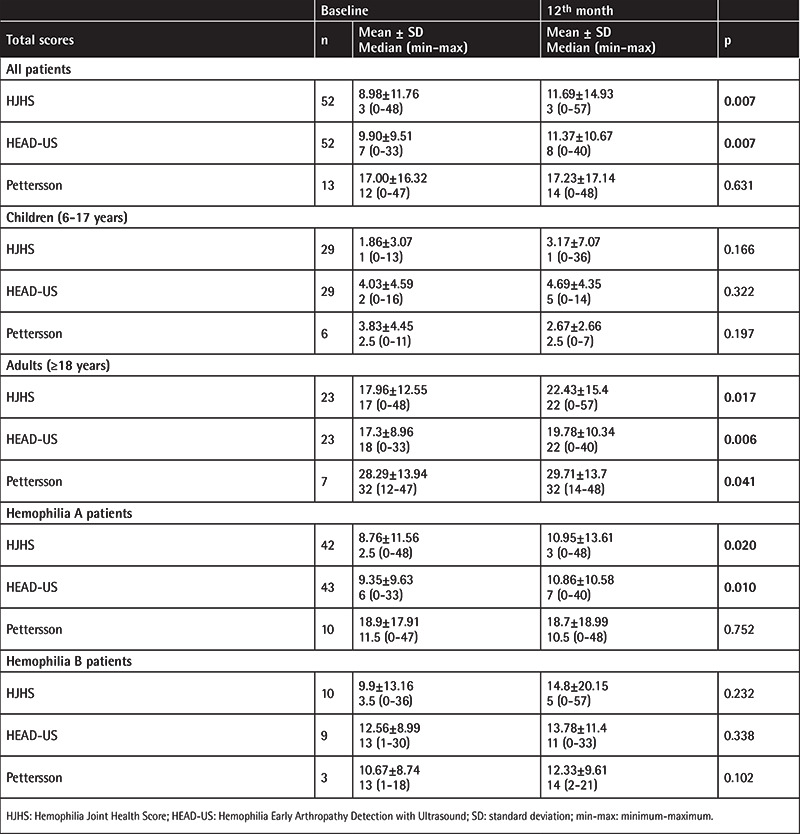
Comparison of the joint scores at the baseline and 12^th^ month.

**Table 7:**
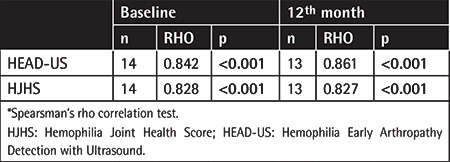
Petterson score and its correlation with HJHS and HEAD-US Scorring system.*

**Table 8:**
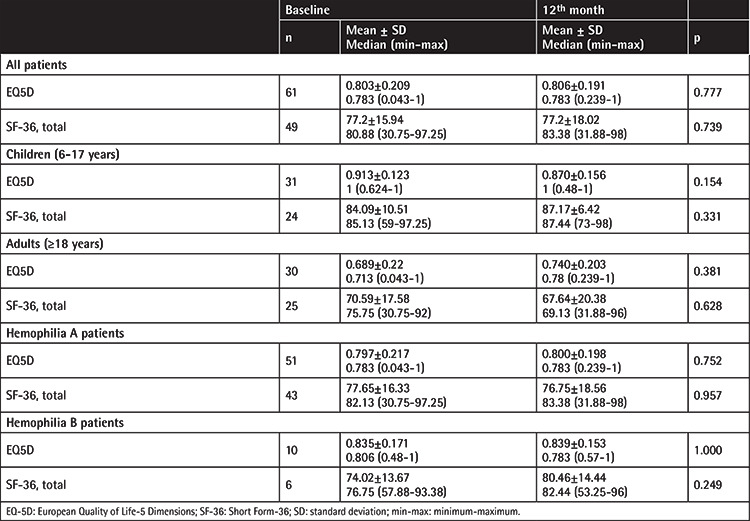
Comparison of the quality of life scores of the patients between the baseline and 12^th^ month.
